# Functional and Immune Modulatory Characteristics of Bone Marrow Mesenchymal Stromal Cells in Patients With Aplastic Anemia: A Systematic Review

**DOI:** 10.3389/fimmu.2022.859668

**Published:** 2022-03-09

**Authors:** Khaled Atmar, Adam J. Tulling, Arjan C. Lankester, Marije Bartels, Frans J. Smiers, Mirjam van der Burg, Alexander B. Mohseny

**Affiliations:** ^1^Department of Pediatric Hematology and Stem Cell Transplantation, Willem-Alexander Children’s Hospital, Leiden University Medical Center, Leiden, Netherlands; ^2^Department of Pediatric Hematology, Wilhelmina Children’s Hospital, University Medical Center Utrecht, Utrecht, Netherlands; ^3^Laboratory for Pediatric Immunology, Department of Pediatrics, Willem-Alexander Children’s Hospital, Leiden University Medical Center, Leiden, Netherlands

**Keywords:** aplastic anaemia (AA), bone marrow failure (BMF), mesenchymal stem (stromal) cell, immunomodulation, hematopoietic regulation, lineage specific differentiation, proliferation, surface marker expression

## Abstract

**Background:**

In most patients with aplastic anemia (AA), the diagnosis is limited to a description of the symptoms. Lack of understanding of the underlying pathophysiological mechanisms causing bone marrow failure (BMF), hampers tailored treatment. In these patients, auto-immune cell-mediated destruction of the bone marrow is often presumed to be the causative mechanism. The status of the bone marrow microenvironment, particularly the mesenchymal stromal cell (MSC) component, was recently suggested as a potential player in the pathophysiology of AA. Therefore, functional, and immune modulatory characteristics of bone marrow MSCs might represent important parameters for AA.

**Objective:**

To conduct a systematic review to evaluate *in vitro* functional properties of MSCs derived from patients with AA compared to healthy controls.

**Methods:**

According to PRISMA guidelines, a comprehensive search strategy was performed by using online databases (Pubmed, ISI Web of Science, Embase, and the Cochrane Library). Studies reporting on phenotypical characterization, proliferation potential, differentiation capacity, immunomodulatory potential, and ability to support hematopoiesis were identified and screened using the Rayyan software tool.

**Results:**

23 articles were included in this systematic review, describing a total of 324 patients with AA and 285 controls. None of the studies identified a significant difference in expression of any MSC surface marker between both groups. However, AA-MSCs showed a decreased proliferation potential, an increased tendency to differentiate into the adipogenic lineage and decreased propensity towards osteogenic differentiation. Importantly, AA-MSCs show reduced capacity of immunosuppression and hematopoietic support in comparison to healthy controls.

**Conclusion:**

We conclude that there are indications for a contribution of MSCs in the pathophysiology of AA. However, the current evidence is of poor quality and requires better defined study populations in addition to a more robust methodology to study MSC biology at a cellular and molecular level. Future studies on bone marrow microenvironment should aim at elucidating the interaction between MSCs, hematopoietic stem cells (HSCs) and immune cells to identify impairments associated with/causing BMF in patients with AA.

## Introduction

Aplastic anemia (AA) is a rare disorder referring to the combination of peripheral pancytopenia and a morphologic/histologic hypocellular bone marrow. AA is characterized by loss of hematopoietic stem cells (HSCs). The term ‘aplastic’ describes the incapability of the bone marrow to produce any of the hematopoietic lineages yet implicates no underlying mechanism or cause. Furthermore, the term ‘anemia’ might be somewhat misleading as patients present with cytopenia in multiple lineages rather than exclusively anemia (1).

To establish the diagnosis of AA, a bone marrow aspiration and biopsy are performed in which, next to a hypocellular marrow, no signs of dysplasia, fibrosis or malignant infiltration are shown. At this stage, often extensive diagnostic evaluation has already been performed to exclude alternative causes for cytopenia, such as various infectious triggers, nutrient deficiencies, medication/toxic related disease, and paroxysmal nocturnal hemoglobinuria (PNH). In addition, bone marrow aspirates undergo cytogenetic analysis by karyotyping (or SNP array) to detect chromosomal aberrations differentiating AA from dysplastic disorders, i.e., myelodysplastic syndrome (MDS). Due to lack of standardization of diagnostic protocols, there can be diagnostic discrepancies at the (inter)national level.

Multipotent mesenchymal stromal cells (MSC), also known as mesenchymal stem cells, are fibroblast-like cells present at different sites and tissues in the body, including the bone marrow, placenta, umbilical cord, dental pulp, and adipose tissue (2). MSCs are known for their capacity to differentiate into cells of various origin and their proposed therapeutic potential. Their distinct properties have led to more than 50.000 scientific publications on this topic in the last three decades as reported by Pittenger et al. (3). To facilitate comparability between studies as well as to enhance uniformity in the processing of MSCs a minimal set of criteria have been established to define a MSC by the Mesenchymal and Tissue Stem Cell Committee of the International Society for Cellular therapy (ISCT). First, MSCs must be plastic-adherent when maintained in standard culture conditions. Second, MSC must express CD105, CD73 and CD90, and lack expression of CD45, CD34, CD14 or CD11b, CD79a or CD19 and HLA-DR surface molecules. Third, MSCs must differentiate into osteoblasts, adipocytes and chondroblasts *in vitro* (**Table 1**) (4).

**Table 1:** Minimal criteria for defining MSCs according to the ISCT.

1. Adherence to plastic in standard culture conditions		
2. Phenotype	Positive (≥95%+) CD105 CD73 CD90	Negative (≥95%+) CD45 CD34 CD14 or CD11b CD79α OR CD19HLA-DR
3. *In vitro* differentiation: osteoblasts, adipocytes, chondroblasts (demonstrated by staining of *in vitro* cell culture)		

Due to their multipotential differentiation capacity, MSCs were long thought to play a role in repair/replacement of damaged tissue. However, in recent years emphasis has shifted more towards the excretory functions of MSCs (3). Within the bone marrow, MSCs are part of the niche, promoting maintenance, proliferation, and differentiation of hematopoietic stem cells (HSC). This is achieved by secretion of soluble factors providing regulatory signals for hematopoietic progenitors specifically (5). Although many properties of the HSC niche have yet to be unraveled, emerging literature has illustrated that MSCs and their adult progeny are able to interact with HSCs directly in a so-called ‘dual stem cell niche’ within perivascular spaces of the bone marrow (BM) (6, 7). Moreover, studies have demonstrated that depletion of CXC chemokine ligand-12 (CXCL12) abundant reticular cells and BM-resident MSCs expressing the marker Nestin, both yielding a modulatory effect on HSC homeostasis, negatively affected absolute HSC count (8, 9). Furthermore, MSCs have been proposed to fulfill an important role in the organization and stabilization of vascular networks in the BM by producing molecules such as Angiopoietin-1, which is pivotal for the organization of HSC niches (10, 11).

In addition to their supportive role, MSCs also exhibit immunomodulatory properties. Immunosuppressive effects of MSCs with regard to cytotoxic- and helper T lymphocytes, natural killer (NK) cells, B lymphocytes have been studied and described extensively (12–15). Additionally, MSCs were shown to inhibit proliferation and differentiation of antigen presenting cells (APC) such as dendritic cells, preventing stimulation of T lymphocytes (16, 17). The interaction between MSCs and immunological effector cells most likely takes places through secretion of immunomodulatory mediators such as transforming growth factor-β1 (TGFβ), interleukin (IL)-10, hepatocyte growth factor, prostaglandin E2 (PGE), indoleamine-2,3-dioxygenase (IDO) and nitric oxide (NO) (18). However, the current understanding is that MSCs are not immunosuppressive by default. Instead, MSCs are thought to develop an immunosuppressive or a pro-inflammatory profile based on the environmental Toll-like receptor (TLR) signals (19). This also suggests that MSCs require some sort of stimulatory signal to employ their immunomodulatory functions. In addition, MSCs can exert their immunomodulatory effects *via* direct cell-to-cell contact involving mechanisms such as FAS/FASL interaction (20).

The growing understanding of MSC physiology allows for novel (therapeutic) applications, including for syndromes and disorders regarding tissue injury and dysregulation of the immune system. For example, graft vs. host disease (GVHD), multiple sclerosis, and diabetes mellitus (21). Moreover, it provides the opportunity to unravel the etiology of a range of illnesses in which MSCs might play a role. AA is regarded as a preeminent example. A condition which could potentially develop when the two main functions of MSCs in the bone marrow are disturbed: HSC support and immunoregulation. Many studies have examined how individual characteristics and functions of MSCs from patients with AA differ from MSCs in healthy individuals. However, to date, no studies have compared these differences in a comprehensive manner. Therefore, we performed a systematic review to compare phenotypic characterization, differentiation capacity, immunomodulatory properties, and ability to support hematopoiesis of MSCs derived from patients with aplastic anemia to healthy controls.

## Methods

This systematic review was conducted according to the 2020 Preferred Reporting Items for Systematic Reviews and Meta-Analyses (PRISMA) statement (22).

### Search Strategy

Studies were identified using multiple electronic databases (Pubmed, ISI Web of Science, Embase and the Cochrane Library). Also, trial registries such as ClinicalTrials.gov were accessed to identify potentially relevant unpublished work. The initial search was performed on the 9th of July 2021. The results were monitored until inclusion on the 5th of October 2021. The main components of the search strategy consisted of the following keywords and related synonyms: “aplastic anemia”, “bone marrow failure” and “mesenchymal stem cells”. Searches were limited to original literature written in English. In addition, a complementary hand-search of potentially relevant studies was performed based on reference lists. If possible, citations were tracked using Google Scholar. Following retrieval of results, Endnote and Rayyan (23) were used to remove any duplicates after which publications were assessed based on title and abstract. Articles deemed highly unlikely to be relevant for this systematic review were expelled from further assessment. Full texts of remaining articles were retrieved and reviewed by two authors independently according to the pre-specified eligibility summarized below. Disagreements on selection were resolved by consensus (**Supplementary 1**).

### Selection Criteria

The following eligibility criteria were applied to select articles:

- Original literature written in English only
- Articles should at least compare human bone marrow mesenchymal stem cells (BM-MSCs) BM-MSCs from AA patients to normal BM-MSCs
- Studied MSCs were unmanipulated, (necessary processing to investigate under culture conditions were allowed)
- MSC properties were (at least) investigated *in vitro*
- At least one of the study outcomes concerns MSC surface marker expression, proliferation, differentiation, immunomodulation, or capacity to support HSCs.
- Utilized test modalities and outcomes of interest relate directly to characterization, proliferation, differentiation, immunomodulation, or capacity to support HSCs.

Studies were excluded when: 1) they assessed the outcomes of interest *in vivo*, but not *in vitro*; 2) MSCs had undergone genetic or potentiating modifications; 3) other cell types were used to generate MSCs (e.g., induced pluripotent stem cells); 4) MSCs were not of human origin; 5) MSCs were derived from other tissues than BM; 6) MSCs were derived from patients and/or controls with other potentially interfering comorbidities; 7) MSCs were derived from patients with one specific type of BMF only; 8) no direct analysis/measurement analysis of MSC characterization, proliferation, differentiation, immunomodulation or capacity to support HSC was performed; 9) published before the year 2000. Other types of literature such as narrative and systematic review articles, book chapters, letters, conference proceedings, and editorials were also excluded.

### Quality Assessment

Methodological quality and risk of bias in studies were assessed independently by two authors (KA and AT) applying a modified version of the Newcastle-Ottawa scale (NOS) for non-randomized studies (24). This tool was most applicable since to date no adequate quality assessment tool has been developed for a non-animal *in vitro* study design. The NOS contains 9 items divided over 3 subjects including: subject selection (scores ranging from 0-6), subject comparability (scores ranging from 0-2), exposure (scores ranging from 0-2). Individual studies do not have an overall quality score assigned to them provided that threshold scores to describe whether a particular study is of low or high quality, respectively, have not yet been validated for the NOS. Consequently, lower quality scores were not an exclusion criterion. Nevertheless, any potential risk factor for bias other than the items in the quality assessment tool was addressed accordingly. Disagreements were resolved by consensus. If necessary, attendance of a third independent reviewer was requested (**Supplementary 2**).

### Data Extraction

Extraction of data from each eligible study was performed by two reviewers (KA and AT). Extracted data consisted of the following study characteristics: name of authors, date of publication, baseline population characteristics (including age, type, and severity of AA), primary/secondary study objective, study groups, prior treatments, test modalities to assess chosen parameters within each outcome domain (MSC morphology, proliferation, differentiation and/or capability to support hematopoiesis) and the corresponding results of these tests. All data was recorded in a table in MS Word (Microsoft Corporation, Redmond, Washington, DC, USA).

## Results

### Characteristics of Included Studies

**Figure 1:**
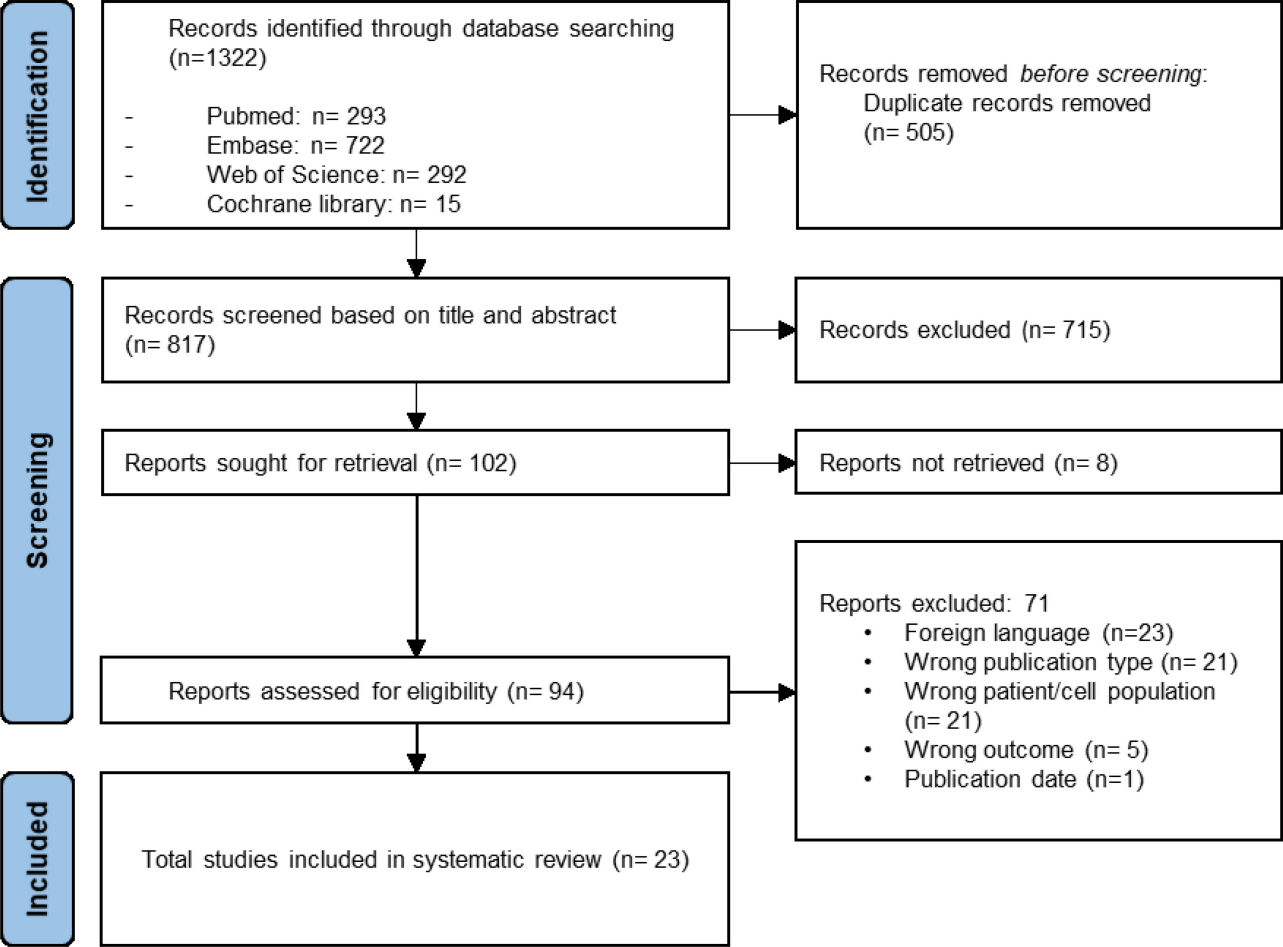
Prisma flowchart illustrating the selection of studies.

**Figure 2:**
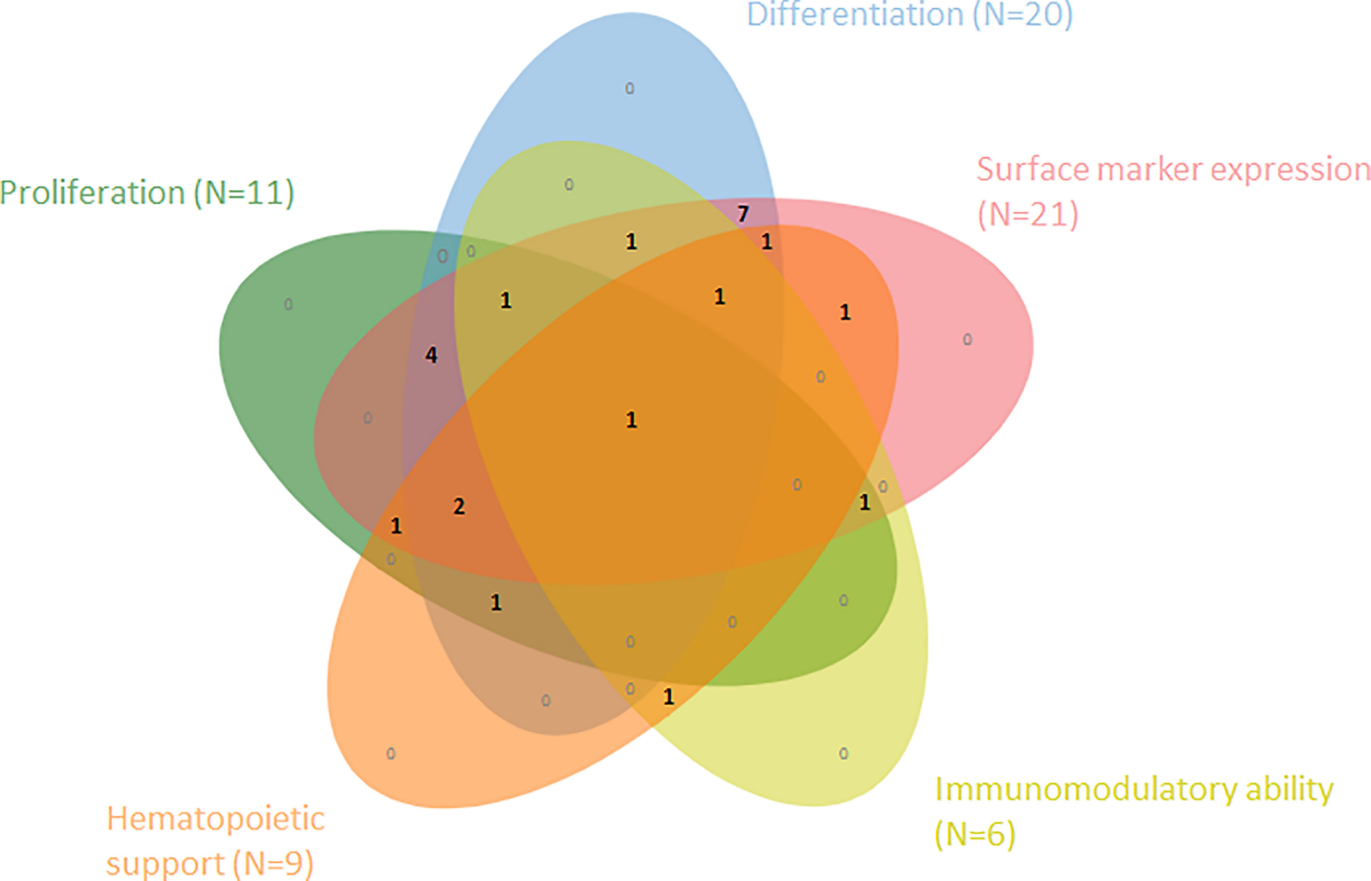
Venn diagram shows the number of articles within this SR that have examined and compared one or more of the five cell characteristics within MSCs from AA-patients vs. healthy controls (N=23).

The systematic literature search yielded a total of 1322 results of which 1220 were removed after duplicate screening and title or abstract screening (**Figure 1**). Of 102 articles assessed in the full-text retrieval and screening phase, we identified 23 studies which met the eligibility criteria described in the methods section. Most studies were conducted in China (57%), followed by India (13%) and Italy (9%). The included studies were geographically representative based on a higher incidence of AA in Asia. As shown in **Table 2** only two studies did not compare cell surface marker expression (30, 45). Proliferation capacity was measured in eleven studies. All but three articles assessed and consequently compared differentiation potential in MSC populations (30, 32, 36). Of the remaining 20 articles, four have investigated MSC differentiation potential in all three lineages (adipocytes, osteoblasts, and chondrocytes). Although Chao et al. (30) did not evaluate surface marker expression nor differentiation potential, these characteristics were analyzed in their previous report, in which the same patient and control populations were used (44). Immunomodulatory- and hematopoietic supporting ability were reported in six and nine articles, respectively. To graphically indicate how many studies have studied each outcome of interest, a Venn Diagram is provided (**Figure 2**) (48). Overlapping shapes correspond with the number of studies that looked at multiple specific characteristics. For example, only one study investigated all five characteristics together.

**Table 2:** *In vitro* properties assessed in control and AA-MSCs.

Author (s)	Year	Groups (n=): controls vs. AA (severity)	Prior treatment (Yes/No/NR)	Proliferation	Differentiation			Surface marker expression	Immunomodulatory ability	Hematopoietic support	Other
					adipo	osteo	chondro				
**Sharma et al.** (25)	2021	5 vs.5 (3 nSAA, 2 SAA)	No	•	•	•	•	•	•		Metabolic activity, appearance with immunofluorescence
**Li, H. et al.** (26)	2021	5 vs. 5	NR			•		•			
**Li, H. et al.** (27)	2020	9 vs. 9	NR		•			•			
**Li, S. et al.** (28)	2020	9 vs. 21 (SAA)	No	•	•	•		•			Apoptotic tendency, PTH levels, T-cell PTH-1R and Wnt factor expression
**Huo et al.** (29)	2020	14 vs. 15 (6 nSAA, 9 SAA)	No	•	•	•	•	•	•		Apoptotic tendency, cellular senescence, cell migration, gene expression profile, appearance with immunofluorescence
**Chao et al.** (30)	2018	5 vs. 5	No						•	•	Apoptotic tendency
**Chaturvedi et al.** (31)	2018	29 vs. 29	NR		•	•		•		•	
**Lu et al.** (32)	2017	19 vs. 28 (24 nSAA, 4 SAA)	NR					•		•	Gene expression profile
**Michelozzi et al.** (33)	2017	7 vs. 8 (2 nSAA, 6 SAA)	Yes (all, prior IST/HSCT)	•	•	•	•	•		•	Appearance with immunofluorescence
**Wei et al.** (34)	2016	12 vs. 12 (7 nSAA, 5 SAA)	No		•			•			
**Cheng et al.** (35)	2015	6 vs. 10	NR		•	•		•			Apoptotic tendency, cellular senescence
**Hamzic et al.** (36)	2015	9 vs. 22 (9 nSAA, 13 SAA)	Yes (9/22, prior IST)	•				•		•	Appearance with immunofluorescence
**Bueno et al.** (37)	2014	7 vs. 9	No		•	•		•	•	•	
**El-Mahgoub et al.** (38)	2014	5 vs.5	NR	•	•	•		•			
**Jiang et al.** (39)	2014	8 vs. 10	NR	•	•	•		•		•	
**Tripathy et al.** (40)	2014	10 vs.10	NR		•	•		•			
**Zhao et al.** (41)	2014	7 vs. 6	No		•	•		•			
**Li, J et al.** (42)	2012	11 vs. 15	No		•	•		•	•		Appearance with immunofluorescence
**Li, J et al.** (43)	2012	20 vs. 21 (SAA)	No	•	•	•		•			Apoptotic tendency, gene expression profile, appearance with immunofluorescence
**Chao et al.** (44)	2010	5 vs. 5 (SAA)	No	•	•	•		•			
**Shipounova et al.** (45)	2009	54 vs. 26 (10 nSAA, 16 SAA)	Yes (17/26 prior treatment)	•	•	•				•	bFGF, ANG-1, VCAM-1, VEGF, BMP, IGF-1 expression
**Xu et al.** (46)	2009	15 vs. 34 (8 nSAA, 19 SAA, 7 vSAA)	Yes (19/34 prior IST)		•	•		•			
**Bacigalupo et al.** (47)	2005	19 vs. 19 (SAA)	Yes (16/19, prior IST)	•	•	•	•	•	•	•	

Bullet point means that this particular outcome in the column is studied in the discussed article. NR, Not reported.

The results of the Newcastle-Ottawa risk of bias assessment ranged from 5 to 9 (**Table 3**). The average score was 6.8. A lower score was mainly related to incomplete or absent characterization of patient cohorts, nonoptimal representativeness of the cases and/or lack of control for age in both study groups, thus increasing the risk for selection bias. This was particularly the case in both articles of Li et al. (26, 27), Cheng et al. (35), and Tripathy et al. (40) which all lacked a comprehensive definition, characterization and representativeness of AA cases. Other factors, such as used products, instruments, processing, and passage numbers of MSCs, were well controlled in all studies. In addition, all studies reported on similar measurement methods of exposure/outcome for all MSCs. Therefore, risk of performance and measurement bias seemed limited.

**Table 3:** Summary of Modified Newcastle-Ottawa scores for included studies.

Authors	Selection					Comparability		Exposure		Total score
	Is the case definition adequate?	Are cases characterized adequately?	Representativeness of the cases	Selection of Controls	definition of controls	Control for important factor (age)	Control for additional factors (substrate, media, growth factors, *in vitro*, passage number)	Ascertainment of exposure	Same method of ascertainment for cases and controls	
	Nr. of stars	Nr. of stars	Nr. of stars	Nr. of stars	Nr. of stars	Nr. of stars	Nr. of stars	Nr. of stars	Nr. of stars	
**Li, J et al.** (43)	1	1	1	1	1	1	1	1	1	**9**
**Li, S. et al.** (28)	1	1	1	1	1	1	1	1	1	**9**
**Bueno et al.** (37)	1	1	0	1	1	1	1	1	1	**8**
**Chao et al.** (30)	1	1	1	0	1	1	1	1	1	**8**
**Li, J et al.** (42)	1	1	0	1	1	1	1	1	1	**8**
**Lu et al.** (32)	1	1	0	1	1	1	1	1	1	**8**
**Wei et al.** (34)	1	1	0	1	1	1	1	1	1	**8**
**Xu et al.** (46)	1	1	1	1	1	0	1	1	1	**8**
**Chao et al.** (44)	1	0	1	0	1	1	1	1	1	**7**
**Hamzic et al.** (36)	1	0	1	1	1	0	1	1	1	**7**
**Huo et al.** (29)	1	0	1	1	1	0	1	1	1	**7**
**Michelozzi et al.** (33)	0	0	1	1	1	1	1	1	1	**7**
**Shipounova et al.** (45)	0	0	1	1	1	1	1	1	1	**7**
**Bacigalupo et al.** (47)	0	0	1	1	1	0	1	1	1	**6**
**Chaturvedi et al.** (31)	1	0	0	0	1	1	1	1	1	**6**
**Cheng et al.** (35)	0	0	0	1	1	1	1	1	1	**6**
**El-Mahgoub et al.** (38)	0	1	0	0	1	1	1	1	1	**6**
**Jiang et al.** (39)	1	0	0	0	1	1	1	1	1	**6**
**Sharma et al.** (25)	1	0	0	1	1	0	1	1	1	**6**
**Li, H. et al.** (27)	0	0	0	0	1	1	1	1	1	**5**
**Li, H. et al.** (26)	0	0	0	0	1	1	1	1	1	**5**
**Tripathy et al.** (40)	0	0	0	0	1	1	1	1	1	**5**
**Zhao et al.** (41)	0	1	0	0	1	0	1	1	1	**5**

Bold values indicate the total number after adding up all the scores for each individual question in each individual study.

### Surface Marker Expression to Define MSCs

Phenotypic characterization is a very important ISCT criterion for the definition of MSCs and was investigated in 21/23 articles. Third passage MSCs were mostly used for analysis of surface marker expression (**Table 4**). The study by Bueno et al. (37) is the only one that fully complies with the marker expression criteria as established by the ISCT. Except for this article, CD79a/CD19 expression has not been examined in any of the other studies. Both studies of Cheng et al. (35) and Jiang et al. (39) reported flowcytometric surface marker expression analysis for all MSCs, however without providing information on any comparative analysis between the groups. None of the studies found a difference in surface marker expression in BM-MSCs derived from patients or controls.

**Table 4:** Assessment of immunophenotyping.

Author	Results	Method of assessment	Passage number	Required surface markers assessed								Other surface markers
				CD105	CD73	CD90	CD34	CD45	CD14/CD11b	CD79a/CD19	HLA-DR	
**Bacigalupo et al.** (47)	No difference	Flow cytometry	3	•		•	•	•			•	CD106, CD166, CD29, CD44
**Bueno et al.** (37)	No difference	Flow cytometry	NR	•	•	•	•	•	•/-	-/•	•	CD44
**Chao et al.** (44)	No difference	Flow cytometry	4	•	•		•	•	•/-			CD44
**Chaturvedi et al.** (31)	No difference	Flow cytometry	3	•	•	•	•	•			•	CD166, CD13
**Cheng et al.** (35)	ND	Flow cytometry	3	•	•	•						
**El-Mahgoub et al.** (38)	No difference	Flow cytometry	3	•		•	•					
**Hamzic et al.** (36)	No difference	Flow cytometry	2 & 4	•			•	•				CD29, CD44, CD166
**Huo et al.** (29)	No difference	Flow cytometry	3	•	•	•	•	•	-/•		•	
**Jiang et al.** (39)	ND	Flow cytometry	NR		•	•	•	•				
**Li, H. et al.** (26)	No difference	Flow cytometry	3-6	•			•	•			•	CD29, CD44
**Li, H. et al.** (27)	No difference	Flow cytometry	3	•			•	•			•	CD29, CD44
**Li, J. et al.** (42)	No difference	Flow cytometry	3	•	•	•	•	•			•	CD29, CD166, CD44, CD49e,
**Li, J. et al.** (43)	No difference	Flow cytometry	3	•	•	•	•	•			•	CD29, CD166, CD44, CD49e, HLA-ABC
**Li, S. et al.** (28)	No difference	Flow cytometry	3	•	•	•	•	•			•	
**Lu, S et al.** (32)	No difference	Flow cytometry	4	•	•	•	•	•	•/•		•	CD13, CD29, CD44, CD49e, CD166, CD31, CD40, HLA-ABC
**Michelozzi et al.** (33)	No difference	Flow cytometry	NR	•	•	•	•	•	•/-		•	CD146, MHC I, MHC II
**Sharma et al.** (25)	No difference	Flow cytometry	3	•	•	•	•	•			•	CD29, HLA-ABC,
**Tripathy et al.** (40)	No difference	Flow cytometry	3	•	•	•	•	•	•/-			
**Wei et al.** (34)	No difference	Flow cytometry	3	•			•	•	•/-		•	CD29, CD44
**Xu et al.** (46)	No difference	Flow cytometry	3	•	•	•	•	•	•/-		•	CD13, CD44, CD29, CD106, CD166,
**Zhao et al.** (41)	No difference	Flow cytometry	NR	•	•		•	•				CD44

Presence of a bulletpoint means that this particular surface marker was studied. In case if two surface markers equally suffice according to the IST criteria and only one of them is studied in an article, a bulletpoint is placed for the studied surface marker, and "–" for the marker that is not studied.NR, Not reported.

### Proliferation

As depicted in **Table 5**, different methods have been used to measure proliferation capacity, including population doubling. Another quantifiable measure for proliferative capacity is the clonogenic potential of cells. This has been examined in 5 studies using the colony forming unit-fibroblast assay (33, 36, 43, 45, 47) (**Table 6**). Whereas the majority of studies (8/11) indicated worse proliferation and clonogenic ability of AA-MSC compared to control-MSC, others (2/11) did not observe a difference (25, 47). In a single study the opposite result was found (45). The latter study showed that AA-MSC maintained their growth capacity beyond the sixth passage while control-MSC stopped growing at this stage. It was also the only study showing that the majority of cell colonies of AA patients were larger in size and higher in quantity as compared to controls.

**Table 5:** Assessment of proliferation potential.

Author	Results	Passage number	Method of assessment
**Chao et al.** (44)	AA-MSC worse average population doubling at each passage AA-MSC worse cumulative population doubling	4-6	Population doublings
**El-Mahgoub et al.** (38)	AA-MSC worse average population doubling at each passage AA-MSC worse cumulative population doubling	1-4	Population doublings
**Hamzic et al.** (36)	No difference in population doubling per passage AA-MS worse cumulative population doubling	2-4	Population doublings
**Huo et al.** (29)	AA-MSC worse average population doubling at 48 hours AA-MSC worse proliferation after 4 and 5 days of culture	3	Population doublings, CCK8 assay (1, 2, 3, 4 and 5 days)
**Jiang et al.** (39)	No difference in proliferation at P0, P1 and P3 AA-MSC worse proliferation after P8	0, 1, 3, 8	Cell count & growth curves (1-14 days for primary culture, 1-9 days for other passages)
**Li, J et al.** (43)	AA-MSC worse proliferation after 4, 6 and 8 days of culture	3	BrdU Cell Proliferation ELISA Kit (0, 2, 4, 6, 8, 10 and 12 days)
**Li, S. et al.** (28)	AA-MSC worse proliferation after 8 and 10 days of culture	3	CCK8 assay (2, 4, 6, 8 and 10 days)
**Michelozzi et al.** (33)	No difference in cumulative population doubling	1-11	Population doublings
**Sharma et al.** (25)	No difference in proliferation potential	3-5	Population doubling time, MTT assay (1, 3, 5, 7 and 14 days)
**Shipounova et al.** (45)	No difference in incrementation time between passages No difference in cumulative cell production from P1-6, thereafter cessation of growth in HD-MSC while continue of growth in AA-MSC	1-61-14	Time to each passage, Cumulative cell production

**Table 6:** Colony forming unit-fibroblast ability (CFU-F).

Author	Results	Passage number	Number of cells per colony	Method of assessment
**Bacigalupo et al.** (47)	No difference in clonogenic potential	0	NR	BMNC seeded in 35-mm wells for 2 weeks, medium change per 3 days, May-Grünwald/Giemsa stained, colonies counted
**Hamzic et al.** (36)	No significant difference in colony-forming potential in BMNC AA-MSC worse colony-forming potential	0, 2	50	BMNC and MSC (P2) seeded in 6-well plates for 2 weeks, medium change per 7 days, Wright’s Giemsa stained, colonies counted
**Li, J et al.** (43)	AA-MSC worse colony-forming potential	1	50	MSC seeded in 6-well plates for 2 weeks, medium change per 3 days, Crystal Violet stained, colonies counted
**Michelozzi et al.** (33)	AA-MSC worse colony forming potential	0	100	MSC seeded in petri dish (size unknown) for 2 weeks, medium change unknown, Giemsa stained, colonies counted
**Shipounova et al.** (45)	Larger size and higher concentration of AA-MSC colonies vs. HD-MSC colonies	0	NR	BMNC seeded in 25cm^2^ flask for 2 weeks, no medium change, Crystal violet stained, colonies counted, and colony size estimated based on digital images

NR, Not reported.

### Differentiation Capacity

**Figure 3:**
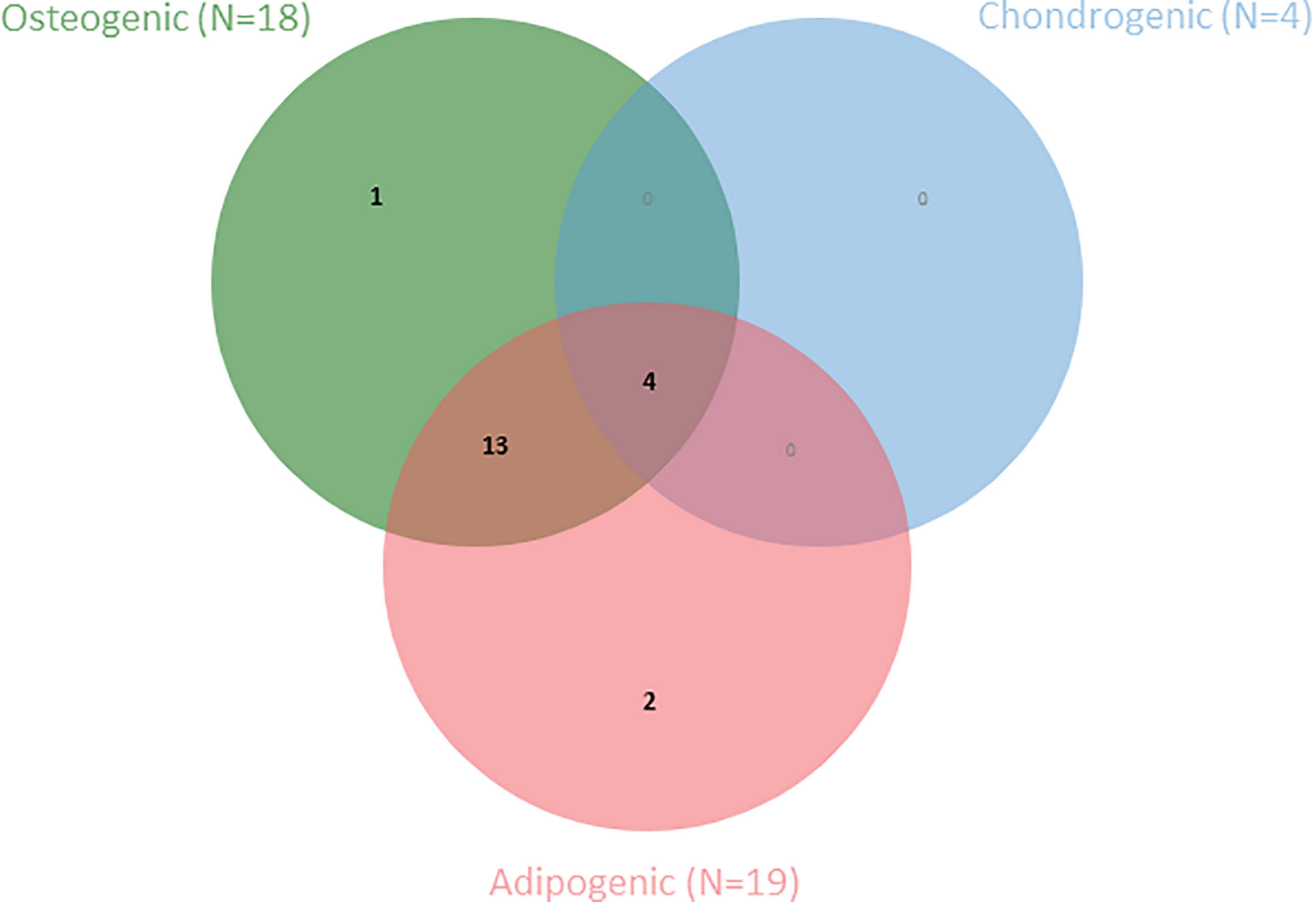
Venn diagram shows the number of articles within this SR that have examined one or more MSC-specific differentiation lineages (N=20).

The final criterion proposed by the ISCT for the definition of MSCs is differentiation capacity towards osteoblasts, adipocytes and chondroblasts *in vitro* (4). Most included studies (N=20) reported on differentiation capacity of the AA-MSCs compared to healthy controls (**Table 7**). Among these articles, Bacigalupo et al. (47) performed a trilineage differentiation assay, and Jiang et al. (39) performed an adipo- and osteogenic differentiation assays for the purpose of MSC quality control, however, without comparing effectiveness of differentiation potential between both groups. A second Venn diagram is presented to depict the number of articles that have studied differentiation towards one or more lineages (**Figure 3**).

**Table 7:** Assessment of differentiation potential.

Author	Results	Passage number	Method of assessment
**Bacigalupo et al.** (47)	ND	NR	Incubation with chondrogenic medium for 14 days; Immunohistochemical and alcian blue staining Incubation with osteogenic medium for 14 days; Alkaline phosphatase activity staining and calcium deposition Incubation with adipogenic medium for 21 days; Oil-Red O staining
**Bueno et al.** (37)	• No difference in differentiation potential	3-5	Incubation with osteogenic medium for 14 days; Alizarin red staining and RT-qPCR (osteocalcin, alkaline phosphatase, osterix) Incubation with adipogenic medium for 14 days; Oil-red O staining and RT-qPCR (PPAR, CEBPα)
**Chao et al.** (44)	• AA-MSC lower osteogenic potential - less intense von Kossa stain and lower ALP activity - lower Cbfa1 expression • AA-MSC lower adipogenic potential - lower Oil-Red O activity - lower LPL expression	3	Incubation with osteogenic medium for 21 days; Alkaline phosphatase activity and von Kossa staining, quantification of alkaline phosphatase activity by spectrophotometry and RT-qPCR (Cbfa1) Incubation with adipogenic medium for 14 days; Oil-red O staining, quantification by spectrophotometry and RT-qPCR (LPL)
**Chaturvedi et al.** (31)	• AA-MSC lower osteogenic potential - less alizarin red staining • AA-MSC higher adipogenic potential - higher density and larger size of lipid droplets/vesicles	3	Incubation with osteogenic medium for 21 days; Alizarin red staining Incubation with adipogenic medium for 18 days; Oil-red O staining
**Cheng et al.** (35)	• AA-MSC lower osteogenic potential - less alizarin red staining - ALPL expression decreased to 68% in AA-patients • AA-MSC higher adipogenic potential - higher density and larger size of lipid droplets/vesicles - Higher PPARγ expression	3	Incubation with osteogenic medium for 21 days; Alizarin red staining, quantification by spectrophotometry and RT-qPCR (BMP2, BMP4, BMP6, BMP7, ALPL) Incubation with adipogenic medium for 21 days; Oil-red O staining, quantification by spectrophotometry and RT-qPCR (PPARγ)
**El-Mahgoub et al.** (38)	• AA-MSC lower osteogenic potential - less mineralization and von-Kossa staining • AA-MSC lower adipogenic potential - less lipid-containing cells and smaller size of lipid droplets	3	Incubation with osteogenic medium for 21 days; von Kossa staining Incubation with adipogenic medium for 14 days; Oil-red O staining
**Huo et al.** (29)	• AA-MSC lower chondrogenic potential - less alcian blue staining - lower ACAN and SOX9 expression • AA-MSC lower osteogenic potential - less alizarin red staining - lower RUNX2 and BGLAP expression • AA-MSC higher adipogenic potential - more lipid droplets - higher ADIPOQ and PPARγ expression	3	Incubation with chondrogenic medium for 14 days; Alcian blue staining and RT-qPCR (ACAN, SOX9) Incubation with osteogenic medium for 14 days; Alizarin red S staining and RT-qPCR (RUNX2, BGLAP) Incubation with adipogenic medium for 14 days; Oil-Red O staining and RT-qPCR (PPARγ, ADIPOQ)
**Jiang et al.** (39)	ND	NR	Incubation with osteogenic medium; von Kossa staining and colony forming unit-osteoblast ability (CFU-O) Incubation with adipogenic medium; Oil red O staining and colony forming unit-adipocyte ability (CFU-Ad)
**Li, H. et al.** (26)	• AA-MSC lower osteogenic potential - less alizarin red staining - higher DNMT1 expression, lower MEG3 expression, lower BMP4 expression	3-6	Incubation with osteogenic medium for 21 days; Alizarin red staining and quantification with spectrophotometry, RT-qPCR/western blot analysis (DNMT1, MEG3, BMP4) and methylation of MEG3 promoter
**Li, H. et al.** (27)	• AA-MSC higher adipogenic potential - more lipid droplets - higher miR-146b-5p expression, lower SIAH2 expression, higher PPARγ expression	3	Incubation with adipogenic medium for; Oil red O staining, RT-qPCR/western blot analysis (miR-146b-5p, SIAH2, PPARγ)
**Li, J et al.** (42)	• AA-MSC lower osteogenic potential - less von Kossa, alizarin red and alkaline phosphatase activity staining • AA-MSC higher adipogenic potential - more Oil red O staining	4	Incubation with osteogenic medium; von Kossa, alizarin red and alkaline phosphatase activity staining Incubation with adipogenic medium; Oil red O staining
**Li, J et al.** (43)	• AA-MSC lower osteogenic potential - less von Kossa, alizarin red and alkaline phosphatase activity staining • AA-MSC higher adipogenic potential - more Oil red O staining	4	Incubation with osteogenic medium; von Kossa, alizarin red and alkaline phosphatase activity staining Incubation with adipogenic medium; Oil red O staining
**Li, S. et al.** (28)	• AA-MSC lower osteogenic potential - less alkaline phosphatase activity staining • AA-MSC higher adipogenic potential - more Oil red O staining	3	Incubation with osteogenic medium for 21 days; Alkaline phosphatase activity staining Incubation with adipogenic medium for 14 days; Oil red O staining
**Michelozzi et al.** (33)	• No difference in differentiation potential	3	Incubation with chondrogenic medium for 21 days; Histological analysis, RT-qPCR (COL2A1, COL10A1, SOX9, ACAN) Incubation with osteogenic medium for 21 days; Alizarin red S staining, RT-qPCR (SPP1, SPARC, ALPL, COL1A2) Incubation with adipogenic medium for 21 days; Oil red O staining, RT-qPCR (FABP4, LPL, PPARγ)
**Sharma et al.** (25)	• No difference in differentiation potential	3	Incubation with chondrogenic medium for 14 days; Alcian blue staining Incubation with osteogenic medium for 28 days; Alizarin Red S staining Incubation with adipogenic medium for 21 days; Oil red O staining
**Shipounova et al.** (45)	• AA-MSC lower osteogenic potential - Among patient MSCs that failed osteogenic differentiation, 80% had SAA • AA-MSC lower adipogenic potential - 1 patient without differentiation, 2 patients changed morphology of MSCs without lipid droplet formation, 3 patients with lipid droplet formation without changed morphology	NR	Incubation with osteogenic medium; Alizarin red staining Incubation with adipogenic medium; Oil red O staining
**Tripathy et al.** (40)	• No difference in osteogenic potential • AA-MSC higher adipogenic potential - higher density and larger size of lipid droplets/vesicles - higher expression of adiponectin and FABP4	3	Incubation with osteogenic medium for 21 days; Alizarin Red S staining, RT-qPCR/western blot analysis (osteopontin) Incubation with adipogenic medium for 18 days; Oil red O staining, RT-qPCR/western blot analysis (adiponectin, FABP4)
**Wei et al.** (34)	• AA-MSC higher adipogenic potential - higher density and larger size of lipid droplets/vesicles at every time point of differentiation - higher FABP4, mTOR, PPARγ, S6K1 and p-S6K1 expression at every time point of differentiation - higher p-mTOR expression	3	Incubation with adipogenic medium for 7, 14 and 21 days; Oil red O staining, western blot analysis/RT-qPCR/immunofluorescence analysis (PPAR-γ, mTOR, p-mTOR, S6K1, p-S6K1, FABP4)
**Xu et al.** (46)	• No difference in osteogenic potential • AA-MSC higher adipogenic potential - no difference in Oil red O staining - lower GATA-2 expression - higher PPARγ expression	3	Incubation with osteogenic medium for 21 days; Silver nitrate staining Incubation with adipogenic medium for 21 days; Oil red O staining, RT-qPCR/western blot analysis (GATA-2, PPARγ)
**Zhao et al.** (41)	• AA-MSC lower osteogenic potential - fewer and smaller mineralized nodes - lower Runx2 expression (P < 0.05) • AA-MSC higher adipogenic potential - more and larger lipid droplets - higher LPL, PPARγ and miR-204 expression	3	Incubation with osteogenic medium for 21 days, Alizarin red staining and quantification with spectrophotometry, RT-qPCR (Runx2) Incubation with adipogenic medium for 21 days, Oil red O staining and quantification with spectrophotometry, RT-qPCR/western blot analysis (LPL, PPARγ, miR-204)

ND, not determinable; NR, not reported.

### Adipogenic Differentiation

Adipogenic differentiation potential was most frequently assessed among the three lineages (19/20 studies). In all studies, the Oil Red O staining method was used to demonstrate adipogenic differentiation. In addition, three studies used a semi-quantitative method by dissolving the staining and measuring the extraction spectrophotometrically (35, 41, 44). As a quantitative method, RT-qPCR and western blot analysis was most frequently used to measure mRNA and protein expression of specific genes directly or indirectly involved in the differentiation process (e.g., PPAR-γ, GATA-2). In 11 studies a higher adipogenic differentiation tendency of AA-MSCs was observed. In contrast, three studies reported lower adipogenic potential among MSCs derived from AA-patients. Of these three studies only one used a quantitative method (44). Finally, no difference was detected between AA-MSCs and control MSCs in three studies (25, 33, 37)

### Osteogenic Differentiation

Osteogenic differentiation was second most frequently studied (18/20 studies). Staining for calcium deposition and mineralization was used as a qualitative measure for osteogenic differentiation. Alizarin red staining was most frequently used followed by von Kossa and alkaline phosphatase activity staining. As with adipogenic differentiation, the same (semi-)quantitative methods were applied to measure osteogenic differentiation capacity. 5/20 studies found no difference in osteogenic differentiation capacity between patients and controls. Three of these studies (3/5) also did not find differences in adipogenic differentiation capacity. On the other hand, 11/20 observed a lower osteogenic differentiation potency in AA-MSCs compared to healthy MSCs.

### Chondrogenic Differentiation

Qualitative methods to measure chondrogenic differentiation potential included Alcian Blue staining in addition to RT-qPCR as a quantitative measurement method. 2/4 studies found no difference in chondrogenic differentiation tendency (25, 33) while one study observed lower chondrogenic potency in AA-MSCs (29). As indicated previously no information could be deduced from the study by Bacigalupo et al. (47).

### Immunomodulation

Immunomodulatory ability is one of the paracrine functions exerted by MSCs. Within the six studies that examined this ability, corresponding methods were used to test this such as the Mixed Lymphocyte Reaction (MLR), Phytohemagglutinin (PHA) stimulation assays, and T-cell differentiation patterns (**Table 8**). In addition, a common method to test immunomodulatory ability was measurement of soluble factors/cytokines produced by effector cells and/or MSCs (e.g., IFN-γ, TNF-α). In general, MSCs are thought to exert an immunosuppressive effect within a pro-inflammatory environment (49, 50). Nevertheless, three studies reported a decreased immunosuppressive ability by AA-MSCs compared to healthy control MSCs as shown by less suppression of T-cell proliferation and higher levels of pro-inflammatory cytokines within AA-MSC co-culture setting (29, 42, 47). Of these studies, Bacigalupo et al. (47) reported that AA-MSCs were less capable of suppressing the inhibitory effect of activated T-cells on hematopoietic colony formation. Furthermore, Huo et al. (29) observed that T cells co-cultured with AA-MSCs were more likely to differentiate into Th1, Th17 and Tc1 cells. This is in line with the findings of Li et al. (43) in which T cells were less likely to differentiate towards regulatory T-cells. In two studies no difference could be detected between both groups (25, 37). In contrast, a single study observed that AA-patient derived MSCs were even more able to suppress proliferation of PHA-activated peripheral bone marrow mononuclear cells (PBMCs) as compared to healthy MSCs (30). Interestingly, in the same study, a higher production of pro-inflammatory cytokines by AA-MSCs was observed in a non-co-culture setting.

**Table 8:** Assessment of immunomodulatory ability.

Author	Results	Method of assessment
**Bacigalupo et al. **(47)	• Less suppression of T-cell proliferation by AA-MSCs and AA-MSC CM • Higher T-cell mediated inhibition of hematopoietic colony formation in AA- MSCs co-cultures • Less suppression of CD38, CD25, CD69, HLA-DR expression on PHA-primed T-cells by AA-MSCs and AA-MSC CM • Less suppression of INF-γ production in co-culture with AA-MSCs	• Mixed lymphocyte reaction; T-cell proliferation • MSC (CM) co-culture with T-cells (PHA); T-cell proliferation, T-cell activation surface markers and IFN-γ production (ELISA), hematopoietic colony formation
**Bueno et al.** (37)	• No difference in immunomodulatory effects	• Mixed lymphocyte reaction; T-cell proliferation, IL-2/TNF-α/IFN-γ production (ELISA) • MSC co-culture with TNF-α/LPS stimulated synovial membrane cells of RA-patients; matrix degrading enzymes (MMP1/MMP8/MMP13 type I collagenase, MMP2 gelatinase, type IV collagenase activities) and TNF-α production (ELISA)
**Chao et al.** (30)	• More suppression of PBMC proliferation by AA-MSCs (P = 0.016) and AA-MSC CM • Higher levels of IL-6, IFN-γ, TNF-α and IL-1β in CM of AA-MSCs • No difference in levels of IL-4, IL-10, IL-17	• MSC (CM) co-culture with PBMCs (PHA); PBMC proliferation • IL-6, IFN-γ, TNF-α, IL-1β, IL-4, IL-10, IL-17 production by MSCs (cytometric bead array immunoassay)
**Huo et al.** (29)	• Less suppression of T-cell activation and differentiation towards Th1, Th17 and Tc1 cells by AA-MSCs	• MSC co-culture with T-cells; T-cell activation (CD25, CD69) and differentiation (Th1, Th2, Th17, Tc1, Tc2)
**Li, J. et al.** (43)	• PHA-cultures: – Less suppression of T-cell proliferation by AA-MSCs – Less suppression of INF-γ and TNF-α production in co-cultures with AA-MSCs – No difference in production of IL-4, IL-10, IL-17 in co-culture with AA-MSCs • MSC single culture: – Lower levels of PGE-2 in CM of AA-MSCs • rhIL-2 cultures: – Lower levels of TGF-β in co-cultures with AA-MSCs – Less promotion of Treg differentiation by AA-MSCs	• MSC co-culture with CD4+ T-cells (PHA); T-cell proliferation and IFN-γ, TNF-α, IL-17, IL-10, IL-4 (ELISA) • PGE2 production by MSCs (ELISA) • MSC co-culture with CD4+ T-cells (rhIL-2); differentiation to Tregs, TGF-β
**Sharma et al.** (25)	• No difference in immunomodulatory effects	• MSC-co-culture with PBMCs (PHA); PBMC proliferation

### Hematopoietic Support

Several mechanisms have been described regarding the supportive role of MSCs within the bone marrow niche, however there is no golden standard technique to analyze this MSC characteristic. This is also reflected by the various methods used in the studies which report on hematopoietic support (N=9) (**Table 9**). Of these studies, 4/9 have used MSC with HSC/bone marrow mononuclear cell (BMNC) co-cultures to test their hypotheses (32, 36, 37, 51). In these studies, hematopoietic support was analyzed by measuring hematopoietic colony formation. Lu et al. (32) and Hamzic et al. (36) observed a decreased colony forming potential of CD34+ cells when cultured with AA-MSCs. In contrast, Bueno et al. (37) and Bacigalupo et al. (47) found no difference in clonogenic potential when both groups were compared. Different from colony forming potential, Chao et al. (30) showed that PBMC proliferation is reduced when co-cultured with AA-MSCs, indicating that AA-MSCs provide less support. Five studies have examined the expression of hematopoiesis-related factors in MSCs, which was highly variable between studies (31–33, 39, 45). Michelozzi et al. (33) reported no difference in gene expression of TGFB1, IL-6, and DDK1 between both groups. Jiang et al. (39) reported a decreased expression of FGF-2 in AA-MSCs, whereas Shipounova et al. (45) studied expression of VCAM-1, ANG-1 and VGEF in adherent cells within long-term bone marrow cultures. After three weeks of culture, the latter study observed an altered gene expression pattern which normalized after another three weeks. Moreover, Chaturvedi et al. (31) stated that specific genes such as G-CSF were significantly higher expressed by AA-MSCs while expression of other genes (e.g., MIP-1α) was lower or did not differ (SCF, TGF-β). The study by Lu et al. (32) was the only study that examined angiogenesis as a well-known paracrine function of MSCs. This was investigated by assessing expression of CD106 (VCAM-1) in MSCs. Next, VEGF production and *in vitro* capillary tube-like formation by MSCs was compared between patients with AA and controls within unsorted MSCs or grouped by presence or absence of the CD106 protein on the MSC. This study showed that CD106+ MSCs had an increased potential for capillary tube-like formation, higher VEGF production and increased hematopoietic colony forming ability. However, expression of CD106 was lower on AA-MSCs compared to healthy MSCs. Consistent with this finding, VEGF production and vasculogenic ability were also reduced in the AA-group. Interestingly, these differences were still present when only CD106+ MSCs were compared between both groups.

**Table 9:** Assessment of hematopoietic supporting activity.

Author	Results	Method of assessment
**Bacigalupo et al.** (47)	• No difference	• MSC long term co-culture with BMNC (LTC-IC)
**Bueno et al.** (37)	• No difference	• MSC co-cultures with UCB-CD34+ cells; proliferation rate, apoptosis frequency, cell cycle analysis and CFU-assay
**Chao et al.** (30)	• Less proliferation of PBMCs in AA-MSCs co-cultures, however no difference when cultured only with AA-MSC CM	• SC co-culture with PBMC; PBMC proliferation
**Chaturvedi et al.** (31)	• RT-qPCR (after LPS stimulation): – Higher expression of TNF-α, G-CSF and SDF-1α expression in AA-MSCs – Lower MIP-1α expression in AA-MSCs – No difference in SCF and TGF-β expression • Measurement in culture supernatant: similar as RT-qPCR, however also no difference in SDF-1α	• RT-qPCR and ELISA; MIP-1α, TNF-α, G-CSF, SDF-1α, SCF, and TGF- expression in LPS induced MSCs
**Hamzic et al.** (36)	• Worse ability to form adherent stromal layers by AA-MSCs at 5 and 6 weeks • Worse colony-forming and proliferation potential of normal CD34+ cells in AA-MSC cross cultures at 6 weeks	• Adherent stromal layer formation • MSC cross-cultures between BM-CD34+ cells (AA/normal) and MSCs (AA/normal; hematopoietic colony formation, non-adherent cell proliferation
**Jiang et al.** (39)	• Lower FGF-2 gene expression in AA-MSCs • Lower FGF-2 levels in BM of AA-patients	• RT-qPCR and ELISA; FGF-2 expression and levels in BM plasma
**Lu, S et al.** (32)	• Lower CD106 and NF-κB expression in AA-MSCs • Lower vasculogenesis ability AA-MSCs • Lower VEGF levels in CM of AA-MSCs • Lower levels of CD34+, CD41+ and CD61+ cells in AA- MSC co-cultures • Less CFU-GM and CFU-mixed cell colonies in AA-MSC co- cultures • Less and smaller CFU-MK colonies in AA-MSC co-cultures	• Illumina sequencing, RT-qPCR, western blot analysis, NanoPro analysis; CD106 and NF-κB expression • Matrigel plug assay; *in vitro* capillary tube-like formation • ELISA; VEGF production by MSCs • MSC co-culture with UCB-CD34+ cells; CFU and CFU-MK assay
**Shipounova et al.** (45)	• Lower expression of ANG-1 and VCAM-1 & higher expression of VEGF after 3 weeks • Normalization of gene expression after 6 weeks	• RT-qPCR; ANG-1, VCAM-1, VEGF in adherent cells of long-term bone marrow cultures
**Michelozzi et al.** (33)	• No difference	• RT-qPCR; TGFB1, IL-6, DDK1

## Discussion

The role of the bone marrow stroma in the pathophysiology of AA is gaining more interest in scientific research. Nevertheless, our study is the first to systematically review and summarize existing literature on *in vitro* characteristics of MSCs of patients with AA compared to healthy controls. In total 23 articles were included in our final analysis. Despite a certain degree of variability, these studies generally showed a decreased proliferation potential in AA-MSCs in addition to alterations in their differentiation capacity such as an increased tendency to differentiate towards adipocytes and a decreased propensity towards the osteogenic lineage. Furthermore, AA-MSCs showed a reduced capacity of immunomodulation and hematopoietic support in comparison to healthy controls. No differences were observed between the two populations regarding expression of MSC-specific surface markers as listed by the ISCT (**Table 10**).

**Table 10:** Summary of results for AA-MSCs per outcome of interest.

Outcomes of interest	Increased (↑)	Decreased (↓)	No difference (=)	Not determinable (ND)
**Proliferation (N=11)**	1	7	3	-
**Differentiation (N=20)**				
Adipogenic (N=19)	11	4	2	2
Osteogenic (N=18)	0	12	4	2
Chondrogenic (N=4)	1	0	2	1
**Surface marker expression (N=21)**	-	-	19	2
**Immunomodulation (N=6)**	1	3	2	‐
**Hematopoietic support (N=9)**	-	6	3	-

Within the included studies, the observed differences were attributed to various hypotheses. Regarding the skewed differentiation patterns observed in AA-MSCs, altered expression of specific transcription factors in MSCs could play an important role. For example, in one study it was seen that a reduced expression of the transcription factor GATA-2 resulted in an increased expression of peroxisome proliferator-activated receptor-γ (PPAR-γ) in AA-MSCs, which strongly correlated with adipogenic differentiation (46). Another suggested factor associated with increased PPAR-γ in MSC of AA patients is an increased expression of mammalian target of rapamycin (mTOR). This hypothesis was supported by two studies demonstrating that adipogenic differentiation can be inhibited by blocking the mTOR signaling pathway (34, 52). Alterations in differentiation behavior of AA-MSCs have also been attributed to changes in expression of certain microRNA (miR), a class of non-coding RNA sequences with post-transcriptional regulatory functions. For instance, Li et al. (27) found an upregulation of miR-146b-5p, which subsequently led to increased expression of PPAR-γ as a result of inhibited downregulation of this protein through SIAH-2 mediated ubiquitination (27). As with adipogenic differentiation, many theories have been proposed to explain impairments in osteogenic differentiation in AA-MSCs. In a more recent study by Li et al. (26), it was shown that expression of MEG3, belonging to another class of noncoding RNAs, was decreased through hypermethylation processes in patients with AA. As a result, expression of BMP4, a known osteogenic factor, was attenuated (26, 35, 53). Increased expression of miRNA-204 and miRNA-144-3p in AA, which are negative regulators of osteogenic factors, Runt-related transcription factor (RUNX) and Tet methylcytosine dioxygenase 2 (TET2), respectively, are also thought to be associated with impaired osteogenesis (41, 54). This is supported by the observation that their silencing rescues MSCs from the impairments in osteogenic differentiation.

An important question is whether the implications of the aberrant cellular characteristics observed *in vitro* can be translated to the *in vivo* situation of patients with AA. In the normal situation MSCs are known to support long-term maintenance and differentiation of hematopoietic progenitor cells by promoting production of cytokines, growth factors, and extracellular matrix proteins in addition to processes maintained through direct cell-to-cell interaction (55–59). Studies have shown that adipogenic and osteogenic differentiation have a reciprocal relationship and when in a balanced state maintain HSCs in homeostasis (60–62). However, an increase in adipogenesis and a decrease in osteogenesis in the bone marrow microenvironment, negatively affects hematopoiesis (63–68). Thus, the findings in this systematic review in terms of aberrant differentiation of MSCs could very likely contribute to the pathophysiology of AA. However, in the aforementioned studies there is no indication that the negative effect of the shifted balance between osteogenesis and adipogenesis on reduced support for hematopoiesis is immune-mediated. Yet, AA is often considered as an immune-mediated BM failure syndrome leading to destruction of HSCs. In line with this hypothesis, it has been demonstrated that MSCs from patients with AA show a reduced capacity of immunomodulation, accompanied by higher T-cell numbers and increased levels of pro-inflammatory cytokines (e.g., TNF-α, IFN-γ) in AA-MSC co-cultures, hence a decreased suppression of T-cell proliferation and corresponding paracrine functions. While it is generally believed that the underlying cause in AA is primarily immunological, the sequence of events is still largely unknown. Does a dysfunctional bone marrow micro-environment lead to auto-reactivity of T-cells or are immune cells causing abnormalities in MSC functioning? Studies included in this review provide clues for both scenarios. Bacigalupo et al. (47) studied clonality of MSCs of seven patients with AA, however they did not detect the presence of any abnormal MSC clone. In addition, the same study showed recovery of T-cell suppressor function in four AA-patients, 1-10 years after hematopoietic stem cell transplantation (HSCT). Both findings support normal MSC function and thereby suggesting a primary problem of patients’ immune system. Nevertheless, the majority of patients with AA do not respond to IST (69) suggesting also different pathophysiological mechanisms or irreversible damage. Considering that MSCs exert influence at multiple levels within the bone marrow, we hypothesize that AA results from a combination of causes involving aberrant stroma failing hematopoietic support as well as promoting a balanced immunological response to damaged stroma. Therefore, it is important to characterize individual cellular abnormalities within stromal cells and their interaction with other local cell types within the bone marrow environment to unravel AA pathogenesis.

### Limitations

Our systematic review encountered certain limitations. Most studies had relatively small sample sizes. In addition, there was a substantial degree of heterogeneity among different studies. This was due to study populations that were not completely similar in severity of AA, sex, and age distribution. As an example, of the 324 patients with AA included in this study, 69 (21,3%) had a non-severe form, 147 (45,4%) were diagnosed with SAA and for the remaining group no information was provided. Moreover, in five of the twenty-three studies, a proportion of the patients had already received treatment by IST or HSCT (n=69). Whether a patient is in remission, refractory to treatment, or in remission might have additionally influenced the results within studies. Similarly, studies also differed in the quality of patient population characterization and exclusion of alternative diagnoses. Apart from heterogeneity in the population of interest, there were also methodological inconsistencies across studies, with variability in cell numbers, materials used and/or differences between, as well as within, techniques used to measure a certain outcome. Finally, it is also important to point out that studies varied in the quality and documentation of the statistical analyses. The limitations mentioned above regarding small sample size, methodological and patient heterogeneity resulted in the fact that a meta-analysis was not possible. This also can explain some conflicting results between different studies or why certain differences were found not to be statistically significant.

The heterogeneity between studies can be explained in part by the unclear disease classification and confusing terminology in AA. As an example, the terms AA and BMF are often used interchangeably, thereby disregarding the hierarchical relationship between these terms. In contrast, in many cases AA is seen as part of a broader collective which is BMF. Thereby, many researchers employ the subsequent distinction between two main categories of AA, namely congenital and acquired AA. The problem with this categorization becomes immediately apparent when known and reliable sources of information provide conflicting information. As an example, the WHO ICD-11 classification makes the distinction between congenital and acquired AA (70). Herein no reference is made to the term BMF. In contrast, UpToDate, uses a different classification in which BMF is divided into the inherited bone marrow failure syndromes (IBMFS) and acquired causes of bone marrow failure of which the latter includes AA (71). Not only do these examples demonstrate the profound obscurity of these commonly used definitions, but in addition they are based on assumptions that are not fully supported by an empiric foundation or that may be considered somewhat outdated as developments in the field progress. It would improve the consistency, homogeneity, and ultimately the internal validity of research if standardized terminology were used in the future with a clear description of the definitions used. Therefore, we underline the essence of a protocolized in-depth diagnostic approach at centers of expertise applying extensive genetic analysis complemented with functional analyses (including telomere length determination) for all patients with suspected BMF as genetic causes are not limited to patients with typical (syndromal) clinical characteristics beyond cytopenia. A higher detection rate of genetic causes for BMF and pre-malignant disorders such as MDS, will result to a better homogenous group of AA for further molecular and cellular analysis to unravel the role of the bone marrow niche for the pathogenesis. Moreover, stratification of the data into IST responders and non-responders will provide more insights regarding the involvement of the immune system in the pathogenesis of AA.

Another important obstacle of investigating bone marrow from BMF patients in general, is that the local disease site is mostly severely affected by the disease, causing destruction at the time of diagnosis so that bone marrow aspirates might not be entirely representative of the bone marrow state but merely represent blood. Thus, it could also provide much relevant information in the future by performing in-depth bone marrow tissue analyses in patients with AA.

## Conclusion

Based on the literature reviewed in this study we can conclude that MSCs from patients with AA are phenotypically similar to those of unaffected individuals, but show fairly consistent differences in terms of proliferation, differentiation, immunomodulation and hematopoietic support. However, the quality of current literature is lacking largely due to the heterogeneity of the selected patient population necessitating caution on the role of MSCs in AA. Future research in which patients suspected for BMF are more thoroughly described and characterized is necessary to identify a uniform population of AA.

## Data Availability Statement

The original contributions presented in the study are included in the article/supplementary material. Further inquiries can be directed to the corresponding authors.

## Author Contributions

KA, MvdB, and AM conceptualized the aims for this systematic review and drafted the manuscript. Searches, quality appraisal and data analysis were carried out by KA and AT. AT, AL, MB, FS, MvdB, and AM reviewed the manuscript and provided critical feedback. All authors contributed to the article and approved the submitted version.

## Conflict of Interest

The authors declare that the research was conducted in the absence of any commercial or financial relationships that could be construed as a potential conflict of interest.

## Publisher’s Note

All claims expressed in this article are solely those of the authors and do not necessarily represent those of their affiliated organizations, or those of the publisher, the editors and the reviewers. Any product that may be evaluated in this article, or claim that may be made by its manufacturer, is not guaranteed or endorsed by the publisher.
