# Supplementary material for: Functional and Immune Modulatory Characteristics of Bone Marrow Mesenchymal Stromal Cells in Patients With Aplastic Anemia: A Systematic Review
Source: Front Immunol. 2022 Mar 9;13:859668. doi: 10.3389/fimmu.2022.859668 (PMC8959635; doi:10.3389/fimmu.2022.859668)
Supplement: Supplementary file 1 [file Table_1.docx]

**Research question:**

How are MSCs from patients with AA different in their characterization, differentiation capacity, immunomodulatory properties and ability to support HSCs compared to MSCs from patients without AA?

## **Pubmed**

Date search: 05-10-2021

("Anemia, Aplastic"[Mesh] OR "aplastic anemia"[tw] OR “aplastic-anemia”[tw] OR "aplastic anemias”[tw] OR “aplastic-anemias”[tw] OR “aplastic anaemia”[tw] OR “aplastic-anaemia”[tw] OR “aplastic anaemias”[tw] OR “aplastic-anaemias”[tw] OR “sAA”[tw] OR “Bone Marrow Failure Disorders"[Mesh] OR “bone marrow failure”[tw] OR “bonemarrow failure”[tw] OR “bone marrow aplasia”[tw] OR “bonemarrow aplasia”[tw] OR “bone marrow hypoplasia”[tw] OR “bonemarrow hypoplasia”[tw] OR “hypoplastic bone marrow”[tw] OR “hypoplastic bonemarrow”[tw])

**Results: 29004**

Action: ADD new component

("Anemia, Aplastic"[Mesh] OR "aplastic anemia"[tw] OR "aplastic-anemia"[tw] OR "aplastic anemias"[tw] OR "aplastic-anemias"[tw] OR "aplastic anaemia"[tw] OR "aplastic-anaemia"[tw] OR "aplastic anaemias"[tw] OR "aplastic-anaemias"[tw] OR "sAA"[tw] OR "Bone Marrow Failure Disorders"[Mesh] OR "bone marrow failure"[tw] OR "bonemarrow failure"[tw] OR "bone marrow aplasia"[tw] OR "bonemarrow aplasia"[tw] OR "bone marrow hypoplasia"[tw] OR "bonemarrow hypoplasia"[tw] OR "hypoplastic bone marrow"[tw] OR "hypoplastic bonemarrow"[tw]) AND ("Mesenchymal Stem Cells"[Mesh] OR "Mesenchymal stem cells"[tw] OR "Mesenchymal stem cell"[tw] OR "mesenchymal stem-cells"[tw] OR "mesenchymal stem-cell"[tw] OR "mesenchymal stromal cells"[tw] OR "mesenchymal stromal cell"[tw] OR "mesenchymal stromal-cell"[tw] OR "mesenchymal stromal-cells"[tw] OR "MSC"[tw] OR "MSCs"[tw] OR "Induced Pluripotent Stem Cells"[Mesh] OR "induced pluripotent stem cells"[tw] OR "induced pluripotent stem cell"[tw] OR "induced pluripotent stem-cells"[tw] OR "induced pluripotent stem-cell"[tw] OR "IPSC"[tw] OR "IPSCs"[tw] OR "IPS cells"[tw] OR "IPS-cells"[tw] OR "IPS cell"[tw] OR "IPS-cell"[tw] OR "hiPSC"[tw])

**Results: 293**

Action: ADD new component

("Anemia, Aplastic"[Mesh] OR "aplastic anemia"[tw] OR “aplastic-anemia”[tw] OR "aplastic anemias”[tw] OR “aplastic-anemias”[tw] OR “aplastic anaemia”[tw] OR “aplastic-anaemia”[tw] OR “aplastic anaemias”[tw] OR “aplastic-anaemias”[tw] OR “sAA”[tw] OR “Bone Marrow Failure Disorders"[Mesh] OR “bone marrow failure”[tw] OR “bonemarrow failure”[tw] OR “bone marrow aplasia”[tw] OR “bonemarrow aplasia”[tw] OR “bone marrow hypoplasia”[tw] OR “bonemarrow hypoplasia”[tw] OR “hypoplastic bone marrow”[tw] OR “hypoplastic bonemarrow”[tw]) AND ("Mesenchymal Stem Cells"[Mesh] OR “Mesenchymal stem cells”[tw] OR “Mesenchymal stem cell”[tw] OR “mesenchymal stem-cells”[tw] OR “mesenchymal stem-cell”[tw] OR “mesenchymal stromal cells”[tw] OR “mesenchymal stromal cell”[tw] OR “mesenchymal stromal-cell”[tw] OR “mesenchymal stromal-cells”[tw] OR “MSC”[tw] OR “MSCs”[tw] OR "Induced Pluripotent Stem Cells"[Mesh] OR “induced pluripotent stem cells”[tw] OR “induced pluripotent stem cell”[tw] OR “induced pluripotent stem-cells”[tw] OR “induced pluripotent stem-cell”[tw] OR “IPSC”[tw] OR “IPSCs”[tw] OR “IPS cells”[tw] OR “IPS-cells”[tw] OR “IPS cell”[tw] OR “IPS-cell”[tw] OR “hiPSC”[tw]) AND ("Hematopoietic Stem Cells"[Mesh] OR “hematopoietic stem cells”[tw] OR “hematopoietic stem cell”[tw] OR “hematopoietic progenitor”[tw] OR “hematopoietic precursor”[tw])

**Results: 131**

Action: Results from second search are subtracted from first search. Resulting articles are screened based on title and abstract.

Action: Returned to the previous 293 results. All were screened based on title and abstract.

## **Embase**

Date search: 05-10-2021

(exp aplastic anemia/ OR "aplastic anemia".mp. OR "aplastic-anemia".mp. OR "aplastic anemias".mp. OR "aplastic-anemias".mp. OR "aplastic anaemia".mp. OR "aplastic-anaemia".mp. OR "aplastic anaemias".mp. OR "aplastic-anaemias".mp. OR "sAA".mp. OR exp Bone Marrow Depression/ OR "bone marrow failure".mp. OR "bonemarrow failure".mp. OR "bone marrow aplasia".mp. OR "bonemarrow aplasia".mp. OR "bone marrow hypoplasia".mp. OR "bonemarrow hypoplasia".mp. OR "hypoplastic bone marrow".mp. OR "hypoplastic bonemarrow".mp.) AND (exp Mesenchymal Stem Cell/ OR "Mesenchymal stem cells".mp. OR "Mesenchymal stem cell".mp. OR "mesenchymal stem-cells".mp. OR "mesenchymal stem-cell".mp. OR "mesenchymal stromal cells".mp. OR "mesenchymal stromal cell".mp. OR "mesenchymal stromal-cell".mp. OR "mesenchymal stromal-cells".mp. OR "MSC".mp. OR "MSCs".mp. OR exp Induced Pluripotent Stem Cell/ OR "induced pluripotent stem cells".mp. OR "induced pluripotent stem cell".mp. OR "induced pluripotent stem-cells".mp. OR "induced pluripotent stem-cell".mp. OR "IPSC".mp. OR "IPSCs".mp. OR "IPS cells".mp. OR "IPS-cells".mp. OR "IPS cell".mp. OR "IPS-cell".mp. OR "hiPSC".mp.)

**Results: 721**

## **Web of Science**

TS=("aplastic anemia" OR "aplastic-anemia" OR "aplastic anemias" OR "aplastic-anemias" OR "aplastic anaemia" OR "aplastic-anaemia" OR "aplastic anaemias" OR "aplastic-anaemias" OR "sAA" OR "Bone Marrow Failure Disorders" OR "bone marrow failure" OR "bonemarrow failure" OR "bone marrow aplasia" OR "bonemarrow aplasia" OR "bone marrow hypoplasia" OR "bonemarrow hypoplasia" OR "hypoplastic bone marrow" OR "hypoplastic bonemarrow") AND TS=("Mesenchymal stem cells" OR "Mesenchymal stem cell" OR "mesenchymal stem-cells" OR "mesenchymal stem-cell" OR "mesenchymal stromal cells" OR "mesenchymal stromal cell" OR "mesenchymal stromal-cell" OR "mesenchymal stromal-cells" OR "MSC" OR "MSCs" OR "induced pluripotent stem cells" OR "induced pluripotent stem cell" OR "induced pluripotent stem-cells" OR "induced pluripotent stem-cell" OR "IPSC" OR "IPSCs" OR "IPS cells" OR "IPS-cells" OR "IPS cell" OR "IPS-cell" OR "hiPSC")

**Results: 291**

## **Cochrane Library**

1. MeSH descriptor: [Anemia, Aplastic] explode all trees
2. ("aplastic anemia" "aplastic-anemia" OR "aplastic anemias" OR "aplastic-anemias" OR "aplastic anaemia" OR "aplastic-anaemia" OR "aplastic anaemias" OR "aplastic-anaemias" OR "sAA" OR "Bone Marrow Failure Disorders" OR "bone marrow failure" OR "bonemarrow failure" OR "bone marrow aplasia" OR "bonemarrow aplasia" OR "bone marrow hypoplasia" OR "bonemarrow hypoplasia" OR "hypoplastic bone marrow" OR "hypoplastic bonemarrow")
3. MeSH descriptor: [Bone Marrow Failure Disorders] explode all trees
4. MeSH descriptor: [Mesenchymal Stem Cells] explode all trees
5. MeSH descriptor: [Induced Pluripotent Stem Cells] explode all trees
6. ("Mesenchymal stem cells" OR "Mesenchymal stem cell" OR "mesenchymal stem-cells" OR "mesenchymal stem-cell" OR "mesenchymal stromal cells" OR "mesenchymal stromal cell" OR "mesenchymal stromal-cell" OR "mesenchymal stromal-cells" OR "MSC" OR "MSCs" OR "induced pluripotent stem cells" OR "induced pluripotent stem cell" OR "induced pluripotent stem-cells" OR "induced pluripotent stem-cell" OR "IPSC" OR "IPSCs" OR "IPS cells" OR "IPS-cells" OR "IPS cell" OR "IPS-cell" OR "hiPSC")
7. #7 (#1 OR #2 OR #3) AND (#4 OR #5 OR #6)

**Results: 15**
