# Supplementary material for: Functional and Immune Modulatory Characteristics of Bone Marrow Mesenchymal Stromal Cells in Patients With Aplastic Anemia: A Systematic Review
Source: Front Immunol. 2022 Mar 9;13:859668. doi: 10.3389/fimmu.2022.859668 (PMC8959635; doi:10.3389/fimmu.2022.859668)
Supplement: Supplementary file 2 [file Table_2.docx]

Quality Assessment Questions

**1) Is the case definition adequate?**

a) Yes, also provides information to specify type of AA/BMF in addition to definition of AA/BMF (acquired, idiopathic, congenital) *

b) Yes, based on internationally acknowledged criteria to define AA (pancytopenia + hypocellular bone marrow)

c) No description

**2) Are cases characterized adequately**

a) Also work-up with WES or comprehensive AA gene panel in addition to basal diagnostics to establish diagnosis of AA/BMF **

b) Also functional assays in addition to basal diagnostics to establish diagnosis AA/BMF *

c) Exclusion of clonal disorders (MDS/AML/PNH) based on bone marrow examination *

d) Only basal diagnostics to establish diagnosis AA/BMF

e) No description

**3) Representativeness of the cases**

a) Consecutive or obviously representative series of cases (e.g., severity) *

b) Potential for selection biases or not stated

**4) Selection of Controls**

a) Healthy donor/volunteer controls *

b) Donor/volunteer control with any form of disease/condition of significance (incl. hematologic diseases without direct effect on BM)

c) No description

**5) Definition of controls**

a) No history of disease (AA) *

b) No description of source

**6) Comparability of cases and controls on the basis of the design or analysis**

a) Study controls for age (most important factor) *

b) Study controls for any additional factor (This criterion could be modified to indicate specific control for a second important factor.) *

**7) Ascertainment of exposure (in our study: characterization, proliferation, differentiation, immunomodulation, support of hematopoiesis)**

a) Uses quantitative measure in addition to qualitative measures for at least one of the main outcomes of interest *

b) Only uses qualitative measures for main outcomes of interest

c) No description

**8) Same method of ascertainment for cases and controls**

a) Yes *

b) No
